# Correlation between phase compatibility and efficient energy conversion in Zr-doped Barium Titanate

**DOI:** 10.1038/s41598-020-60335-5

**Published:** 2020-02-26

**Authors:** Maike Wegner, Hanlin Gu, Richard D. James, Eckhard Quandt

**Affiliations:** 1 Institute for Materials Science, Kiel University, Kiel, 24143 Germany; 2 Aerospace Engineering and Mechanics, University of Minnesota, Minneapolis, 55455 USA

**Keywords:** Materials for devices, Ceramics, Ferroelectrics and multiferroics, Phase transitions and critical phenomena

## Abstract

Recent demonstrations of both heat-to-electricity energy conversion devices and electrocaloric devices based on first-order ferroelectric phase transformations identify the lowering of hysteresis and cyclic reversibility of the transformation as enabling criteria for the advancement of this technology. These demonstrations, and recent studies of the hysteresis of phase transformations in oxides, show that satisfying conditions of supercompatibility can be useful for lowering hysteresis, but with limitations for systems with only a few variants of the lower symmetry phase. In particular, it is widely accepted that in a classic cubic-to-tetragonal phase transformation, with only three tetragonal variants having only six twin systems, tuning for improved crystallographic compatibility will be of limited value. This work shows that, on the contrary, the tuning of lattice parameters in Ba(Ti_1-x_Zr_x_)O_3_ for improved crystallographic compatibility, even at low doping levels of Zr (x ≤ 0.027), give significant improvement of transformation and ferroelectric energy conversion properties. Specifically, the transformation hysteresis is lowered by 25%, and the maximum value of the polarization/temperature ratio dP/dT at the phase transformation is increased by 10%.

## Introduction

Recently, new devices for the direct (i.e. without a separate electrical generator) conversion of heat to electricity have emerged based on first order ferroelectric phase transformations^1,2^. These devices are designed to harvest energy from small temperature differences in the environment, from industrial sources, or from computers and digital devices. From a materials science perspective the main challenges facing the development of this technology, as identified in these papers, are the management of leakage, the cyclic reversibility of the transformation, and the minimization of hysteresis. The latter, which is of interest here, is identified as the main source of loss in this application.

In metals, most prominently in TiNi-based shape memory alloys, an understanding of the factors affecting reversibility and hysteresis is quite far advanced. For a high cyclic reversibility it is well-accepted^3^ that having a high to low symmetry transformation with a group-subgroup relation, small volume change, and the existence of many low energy austenite/martensite interfaces are desirable. As an example of the influence of these factors, no reasonable shape memory effect has been demonstrated using any metal with a cubic-to-tetragonal phase transformation in polycrystalline form. As demonstrated in slightly Ni-rich TiNi alloys, a fine array of suitable coherent precipitates is also highly desirable, as is a fine, but not too fine, grain size^3,4^. The most dramatic improvements in cyclic reversibility, and also thermal hysteresis, have been achieved by tuning the lattice parameters to satisfy conditions of supercompatibility^5–8^. Supercompatibility refers to special conditions on the lattice parameters that eliminate stressed transition layers between phases. The simplest condition of supercompatibility, *λ*_2_ = 1, implies the existence of a perfect unstressed interface between phases. In cubic-to-tetragonal phase transformations, with lattice parameters *a*_0_ (cubic) and *a*, *c* (tetragonal), the condition *λ*_2_ = 1 is simply *a* = *a*_0_. Thus, the cubic-to-tetragonal transformation is so inflexible that the condition *λ*_2_ = 1 contradicts a zero volume change.

In less rigid systems, by adjusting the lattice parameters to satisfy conditions of supercompatibility, it is possible to design materials with highly improved fatigue behaviour and dramatically lowered thermal hysteresis as was shown for Zn45Au30Cu25^9,10^ and Ti54.7Ni30.7Cu12.3Co2.3^3^. It is important to note that small deviations from perfect supercompatibility already result in dramatic degradation of these functional properties, thus requiring careful attention to small changes in composition^8^.

The possible application of conditions of supercompatibility to diffusionless phase transformations in oxides has recently generated significant interest^11–13^. The case of oxides is viewed as most challenging in view of their inherent brittleness, the relative scarcity of high to low symmetry transformations, and the presence of relaxor effects that tend to smear out first order transformations in nonstoichiometric compounds^13^. Nevertheless, it is demonstrated^11,12^ that conditions of supercompatibility have a very significant effect on thermal hysteresis.

In this work barium titanate-based compounds are chosen as the model system as they were used in recent demonstrations of energy conversion devices^1,2^ and they show overall attractive properties for electrocaloric applications^14–16^ and as lead-free piezoceramics. Thus, in regard to the importance of barium titanate to energy conversion and electrocaloric devices, its general rigidity (3 variants, 6 twin systems), and its status as a lead-free perovskite, it is of high interest to examine conditions of supercompatibility, in particular the condition *λ*_2_ = 1, in cubic-to-tetragonal oxide systems. To the best of our knowledge, the tuning of lattice parameters in a cubic-to-tetragonal transformation to achieve conditions of compatibility has not been done for any material, metal or oxide. Barium titanate-based compounds also allow for a wide-range of partial substitution of ions with appropriate electronegativity on all crystallographic positions of the perovskite structure^17^. We focus on precisely tuning the Zr-content in Ba(Ti_1-x_Zr_x_)O_3_ with x < 0.03 to identify compositions which fulfil the supercompatibility conditions. Ba(Ti_1-x_Zr_x_)O_3_ in this compositional range shows first order phase transformation for all three transitions, cubic-tetragonal, tetragonal-orthorhombic and orthorhombic-rhombohedral^18,19^, which are also inducible by applying an electric field^20^ and thereby exhibit an electrocaloric effect^21^. These phase transformations show a thermal hysteresis, which has also big influence on the electrocaloric response^14^. For a high reversibility, a high coefficient of performance^1^ and low fatigue the thermal hysteresis should therefore be as low as possible.

In this paper we study the influence of fine-doping on the crystallographic compatibility of the cubic and tetragonal phase of barium titanate and the connection between the thermal hysteresis as well as of functional properties and the degree of compatibility between the cubic and the tetragonal phase.

## Theory

Based on the geometrically nonlinear theory of martensite, Zhang and James found several conditions that have an effect on the minimization of the hysteresis and the reversibility of the phase transformation^22^. The conditions have their origin in the crystal structure and the lattice parameters of the participating phases. The simplest condition of supercompatibility of the participating phases is *λ*_2_ = 1, where *λ*_1_ ≤ *λ*_2_ ≤ *λ*_3_ are the ordered eigenvalues of the transformation stretch matrix *U* (see below). Here, as noted above, the terminology *supercompatibility* refers to conditions on lattice parameters that eliminate stressed transition layers between phases. There is an increasing evidence for metals that *λ*_2_ = 1 largely influences hysteresis^3,23,24^. Stronger conditions of supercompatibility called the *cofactor conditions* also increase the fatigue life^3^.

The transformation stretch matrix can be deduced from the group-subgroup relation of the point group symmetries of the two considered phases. The transformation from one lattice to the other during the phase transition can be represented by a linear transformation *F*, which has a polar decomposition *F* = *RU*, where *R* is a 3×3 rotation matrix and *U* is the transformation stretch matrix^24^. Austenite and single-variant martensite form a perfect planar interface without the occurrence of a transition layer if and only if *λ*_2_ = 1^24^. Due to the fact that *λ*_2_ depends only on the crystal structure and the lattice parameters, it is possible to achieve *λ*_2_ = 1 by compositional variations, e.g. by doping with various concentrations^3^.

The cubic-to-tetragonal case in barium titanate based compounds is a particularly easy case to seek conditions of supercompatibility. For this material the cubic, high-temperature paraelectric phase is referred to as the austenitic phase, as this structure has the highest symmetry and can be compared with metallic austenitic structures, while the tetragonal, low-temperature phase is the martensitic phase with the lower symmetry and twinned structure, where the single domains are connected to the martensitic variants. From the lattice parameters close to the phase transition the middle eigenvalue can be calculated by using the transformation stretch matrix

1 $${U}_{c-t}=(\begin{array}{ccc}\frac{a}{{a}_{0}} & 0 & 0\\ 0 & \frac{a}{{a}_{0}} & 0\\ 0 & 0 & \frac{{\rm{c}}}{{a}_{0}}\end{array})$$

where *U*_*c-t*_ is the transformation stretch matrix for the cubic to tetragonal transition, *a* is the lattice parameter a of the tetragonal phase, *c* is the lattice parameter c of the tetragonal phase and *a*_0_ is the lattice parameter a of the cubic phase. Clearly, the middle eigenvalue of *U*_*c-t*_ is ${\lambda }_{2}=\frac{a}{{a}_{0}}$.

The condition *λ*_2_ = 1 is also part of the cofactor conditions. In addition to *λ*_2_ = 1 the cofactor conditions also include an equality and an inequality regarding the twinning system which occurs in the martensitic phase. Physically, the cofactor conditions mean that the material can form austenite/twinned martensite interfaces with any volume fraction of the two variants of martensite^25^. The cofactor conditions can be simplified to an equation of the form det(*G*_*f*_*-I*) = *q*(*f*) = 0 for the volume fraction *f* of the twin pair that participates in the austenite/martensite interface. The derivation and definition of the simplified version of the cofactor conditions can be found in Theorem 2 of^25^. For the cubic to tetragonal phase transition the formulas regarding the calculation of q(f) are described in detail in the supplemental section. For 0 ≤ *f* ≤ 1, the function *q*(*f*) is quadratic and is symmetric about *f* = *1/2*. According to^3^ the cofactor conditions are

CC1 $$q(0)=0\leftrightarrow {\lambda }_{2}=1,$$

CC2 $$q{\prime} (0)=0\leftrightarrow a\cdot Ucof({U}^{2}-I)n,$$

CC3 $$tr{U}^{2}-det{U}^{2}-\frac{{a}^{2}{n}^{2}}{4}-2\ge 0.$$

When the cofactor conditions are fulfilled the material also has the possibility to form interfaces with zero elastic energy austenite/twinned martensite interfaces with any volume fraction of the twin variants and the austenitic phase^3^.

## Results and Discussion

The influence of the amount of Zr on the phase transition temperatures and thermal hysteresis is investigated by Differential Scanning Calorimetry (DSC) with a heating rate of 10 Kmin^−1^. The results of the DSC measurements including the values for the thermal hysteresis *ΔT* as well as the corresponding Curie-temperature *T*_*c*_ are shown in Table 1.

**Table 1:** DSC results of Ba (Ti_1-x_Zr_x_)O_3_ samples for the cubic-to-tetragonal phase transition.

x	T_c_ [°C]	P_s_ [°C]	P_f_ [°C]	F_s_ [°C]	F_f_ [°C]	ΔT [K]
0.006	133.4	122.5	126.6	121.4	117.3	5.21
0.009	130.8	121.3	124.1	119.8	117.2	4.25
0.013	129.3	119.0	121.1	118.0	114.7	4.19
0.017	127.9	117.1	120.7	117.0	112.9	3.93
0.027	124.8	113.5	117.2	113.4	108.7	4.24

The values of *P*_*s*_, *P*_*f*_, *F*_*s*_ and *F*_*f*_ were determined using the tangent method where *P*_*s*_ and *P*_*f*_ are the start and finish temperatures of the paraelectric cubic phase, respectively, and *Fs* and *F*_*f*_ are the start and finish temperatures of the ferroelectric tetragonal phase, respectively. *ΔT* = 1/2(*P*_*s*_ + *P*_*f*_-*F*_*s*_-*F*_*f*_) was used to calculate the thermal hysteresis. The Curie-temperature *T*_*C*_ was determined for a frequency of 1 kHz.

**Figure 1:**
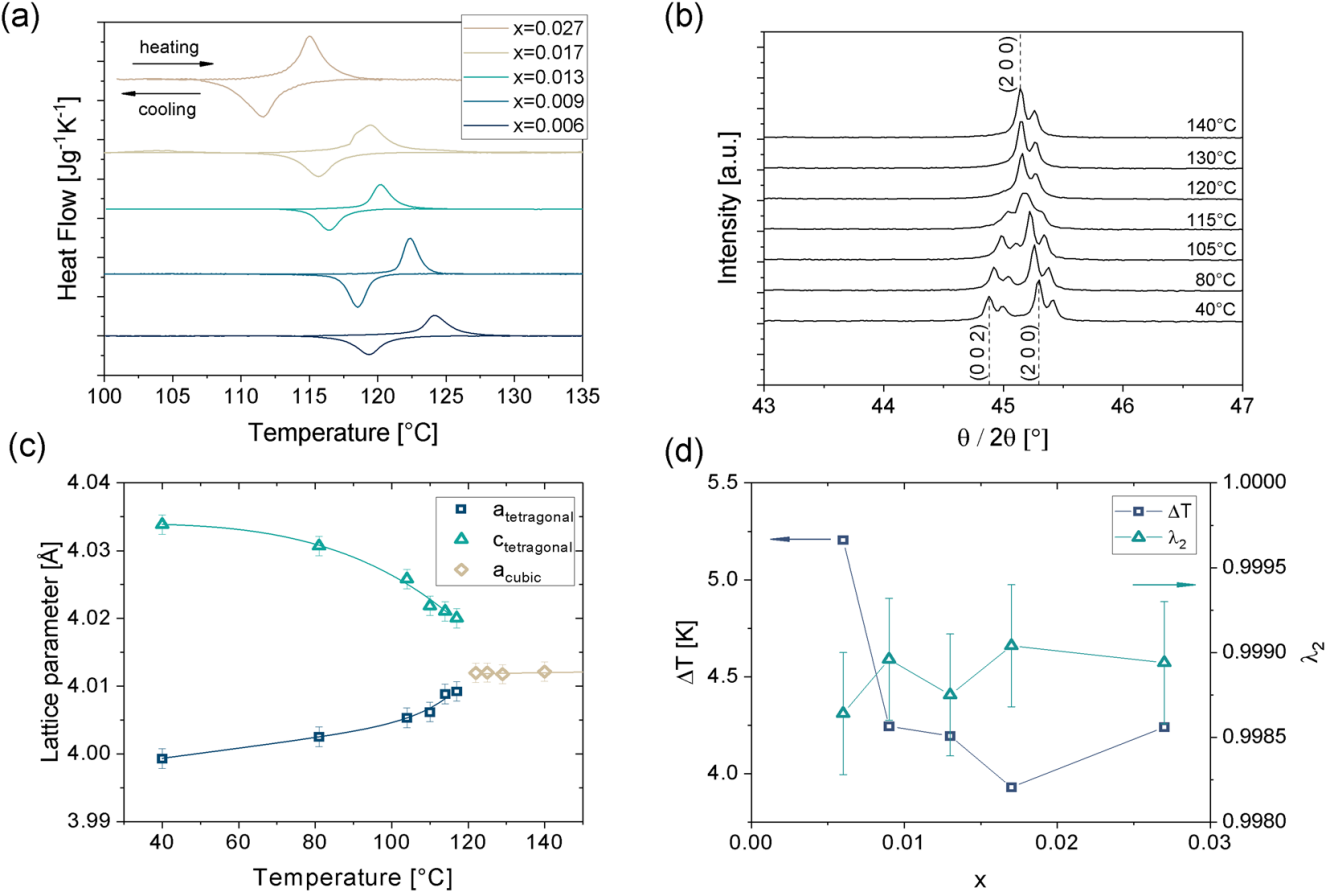
Results of thermal and crystallographic analysis (**a**) DSC measurements of Ba(Ti_1-x_Zr_x_)O_3_ samples with various values for x of forward and reverse cubic-tetragonal phase transition. (**b**) Temperature dependent diffractograms of Ba(Ti_0.983_Zr_0.017_)O_3_ reduced to the (0 0 2)(2 0 0) and (2 0 0) reflections at various temperatures between 40 °C and 140 °C indicating the cubic-to-tetragonal phase transition. (**c**) Temperature dependent lattice parameters of tetragonal and cubic phase for Ba(Ti_0.983_Zr_0.017_)O_3_ with error bars equal to a standard deviation of 0.036%. (**d**) Dependence of the thermal hysteresis and the middle eigenvalue of the transformation stretch matrix on the amount of Zr in the system Ba(Ti_1-x_Zr_x_)O_3_.

The corresponding heat flow curves are depicted in Fig. 1a. Obviously the transition temperatures are very sensitive regarding changes in the composition of the material. Already for very small changes of amounts of Zr ions on the Ti-sites the transition temperatures are lowered. The tendency of decreasing transition temperatures with increasing amount of Zr ions on the Ti-sites is well known and described elsewhere^17^. Besides the transition temperature also the thermal hysteresis of the cubic-tetragonal phase transition is affected by the small doping amounts. Especially comparing the compositions of Ba(Ti_1-x_Zr_x_)O_3_ with x = 0.006 and x = 0.017 indicates on a change of the thermal hysteresis from *ΔT* = 5.21 K to *ΔT* = 3.93 K, respectively.

The X-ray diffraction (XRD) measurements were taken in the temperature range of 40 to 140 °C, a typical example of the course of diffractograms over temperature is shown in Supplementary Fig. S1. The phase transition from tetragonal to cubic can be clearly identified from the vanishing peak splitting of e.g. the (0 0 2)(2 0 0) peak, see Fig. 1b. The side peaks on the right side of the main peaks are related to the Kα_2_-line of the Cu K-alpha spectral line. For the tetragonal phase one can clearly distinguish two peaks representing the (2 0 0) and (0 0 2) reflection while for the cubic phase the two peaks are merging to one single peak representing the (2 0 0) reflection.

In order to perform the structural refinement X-ray powder diffraction data is needed as a starting point for the simulation of the pattern. For the cubic and the tetragonal phase data sets of Mahmood *et al*. were used, including the coordinates of the atomic sites^26^. The peak shape was defined by the modified Thompson-Cox-Hastings pseudo-Voigt function^27^. The occupancy of the Ti-site has been modified according to the corresponding amount of zirconium. In Supplementary Fig. S2 the structural refinement is shown for Ba(Ti_0.983_Zr_0.017_)O_3_ at a temperature of 80 °C.

The refinement was performed for each concentration for several temperatures in order to identify the temperature dependent lattice parameters, which are shown in Fig. 1c and Supplementary Fig. S2. The error bars are referring to the statistical error of measurements, which has been determined by evaluation of 10 individual recorded diffractograms. The standard deviation has been calculated to be equal to 0.036%.

The dependence of the middle eigenvalue on the amount of zirconium have been calculated with the transformation stretch matrix in Eq. (1). According to the theory the closest value of *λ*_2_ to unity is expected to come along with the lowest thermal hysteresis. The results of the middle eigenvalue and the thermal hysteresis *ΔT* in dependence of the amount of Zr on the Ti-site is depicted in Fig. 1d. With increasing Zr-content the thermal hysteresis is decreasing, while the course of the middle eigenvalue tends to get closer to one with increasing Zr-amount. The error bars of the middle eigenvalue have been calculated by using the maximum and minimum values for the lattice parameters *a*, *c* and *a*_0_ considering the determined statistical error of 0.036%. The interconnection between the minimum of thermal hysteresis and the proximity of the middle to unity is perceivable for x = 0.017. One might expect that, with a monotonic change of the lattice parameters with the amount of Zr, the middle eigenvalue would also change monotonically. However, this is indeed not the case as the middle eigenvalue is equal to the quotient of *a* over *a*_0_, where *a* and *a*_0_ are monotonically dependent of the amount of Zr and also dependent of the temperature. Thus this function has a local maximum, which is in this case at x = 0.017.

**Figure 2:**
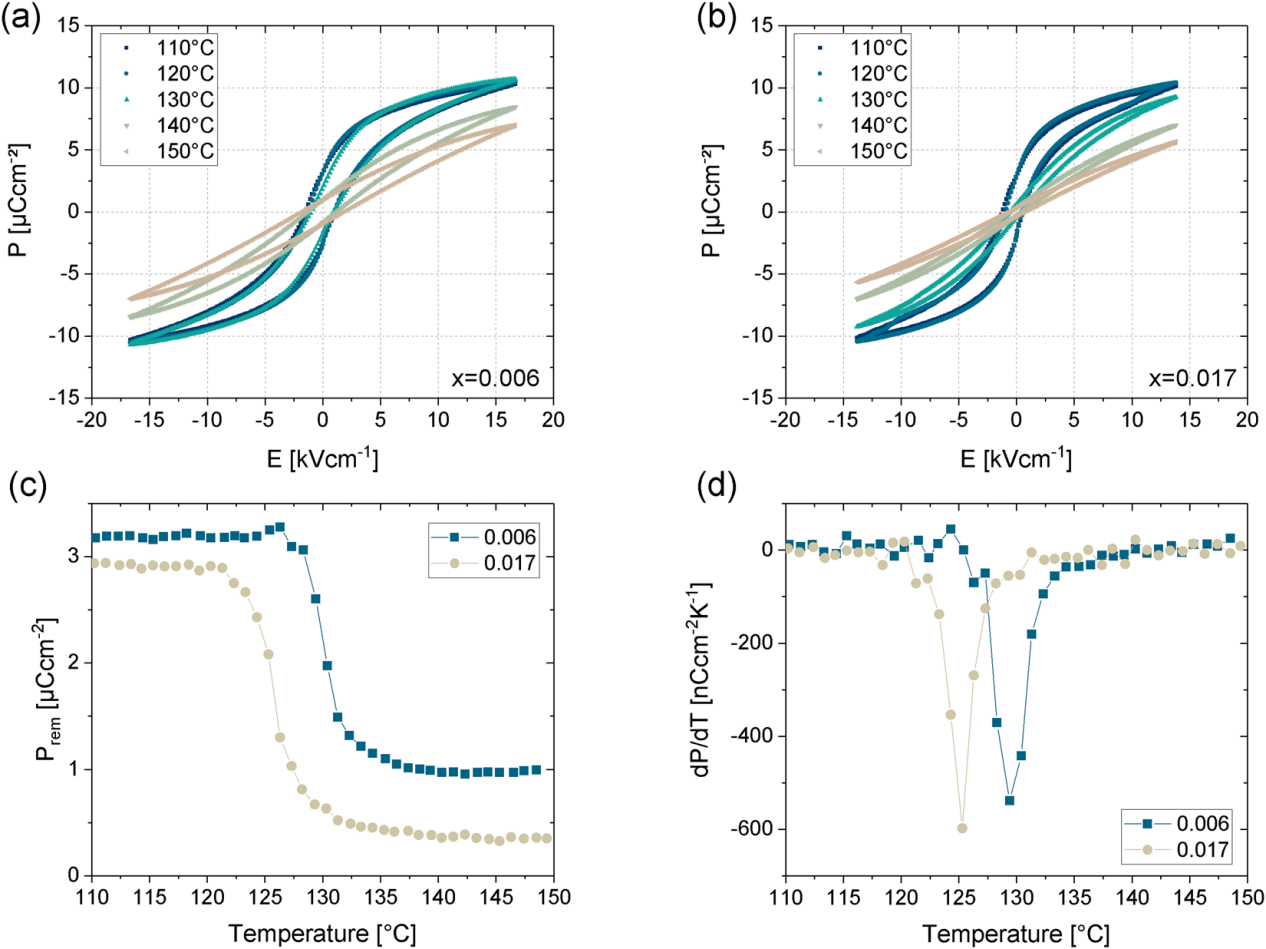
Electrical characterization in regard to the temperature dependent behaviour of polarization. Temperature dependent *P*(*E*)-hysteresis loops for Ba(Ti_1-x_Zr_x_)O_3_ with (**a**) x = 0.006 and (**b)** x = 0.017 and the corresponding temperature dependent (**c**) remnant polarization and (**d**) dP/dT.

For the compositions of Ba(Ti_1-x_Zr_x_)O_3_ with x = 0.006 and x = 0.017 the electrical properties have been characterized at a frequency of 10 Hz (see Fig. 2a,b), as those were the samples with the worst and the best thermal hysteresis corresponding to a middle eigenvalue further from or closer to one. A clear change from ferroelectric to paraelectric behaviour can be detected by passing the transition temperature from cubic to tetragonal phase. The high temperature phase shows an almost linear dependence of polarization on electric field, while for the tetragonal phase below *T*_*C*_, a typical hysteresis is seen in Fig. 2a,b.

By visualizing the dependence of the remnant polarization on the temperature (see Fig. 2c) the phase transition is clearly detectable. In accordance with the DSC characterization the transition temperature decreases with increasing amount of Zr. The remnant polarization of the sample with x = 0.017 is slightly lower compared to the sample with x = 0.006, but the absolute change of remnant polarization *ΔP = P*_*rem,ferroelectric*_* − P*_*rem,paraelectric*_ for the sample with x = 0.017 (Δ*P* = 2.52 µCcm^−2^) is larger than that for the sample with x = 0.006 (Δ*P* = 2.22 µCcm^−2^). By taking the derivative of the temperature dependent remnant polarization (see Fig. 2d), the rate of change of polarization with temperature can be determined, which is related to the electrocaloric effect based on the Maxwell relationship ${(\frac{\partial S}{\partial E})}_{T}={(\frac{\partial P}{\partial T})}_{E}$. By changing the external electric field under adiabatic conditions, the reversible electrocaloric change in temperature $\Delta T$ is typically described by

2 $$\Delta T=-\,{\int }_{{E}_{1}}^{{E}_{2}}\frac{T}{c(E,T)\rho }{(\frac{\partial P}{\partial T})}_{E}dE$$

where *T* is the temperature, *c(E,T)* is the heat capacity, *ρ* is the density and *P* is the polarization. Equation 2 indicates that a large change of polarization with temperature *(∂P/∂T)*_*E*_ over the interval of electric field values *(E*_1_*,E*_2_) is required in order to gain a high temperature change based on the electrocaloric effect^28^. Comparing the samples with x = 0.006 and x = 0.017, where x = 0.017 has the lower thermal hysteresis and a middle eigenvalue of the transformation stretch matrix closest to one, a maximum value of *dP/dT* of −538 nCcm^−^²K^−1^ is reached for x = 0.006, while for x = 0.017 a value of −597 nCcm^−^²K^−1^ is obtained, which gives an enhancement of 10% compared to the sample with the lower amount of Zr. This is strongly suggesting that the enhancement is related to the improved compatibility of the participating phases. Besides the maximum value, also the FWHM of the peaks is improved from 2.8 K for x = 0.006 to 2.1 K for x = 0.017.

## Analysis of Crystallographic Compatibility

The experimental results shown before admit a surprising interpretation, especially in view of the fact that it is a cubic-to-tetragonal transformation. Even though the transformation is becoming closer to second-order with increasing Zr content, a local minimum of hysteresis occurs at x = 0.017, where also *λ*_2_ is closest to one. It is therefore instructive to examine more closely the conditions of supercompatibility in this case.

As already summarized above, the cofactor conditions are the vanishing of the quadratic function *q(f)* that arises in the crystallographic theory of martensite. The generic situation (with typical reversible martensites) is that *q(f) = 0* has two solutions f* and (1 - f*).

**Figure 3:**
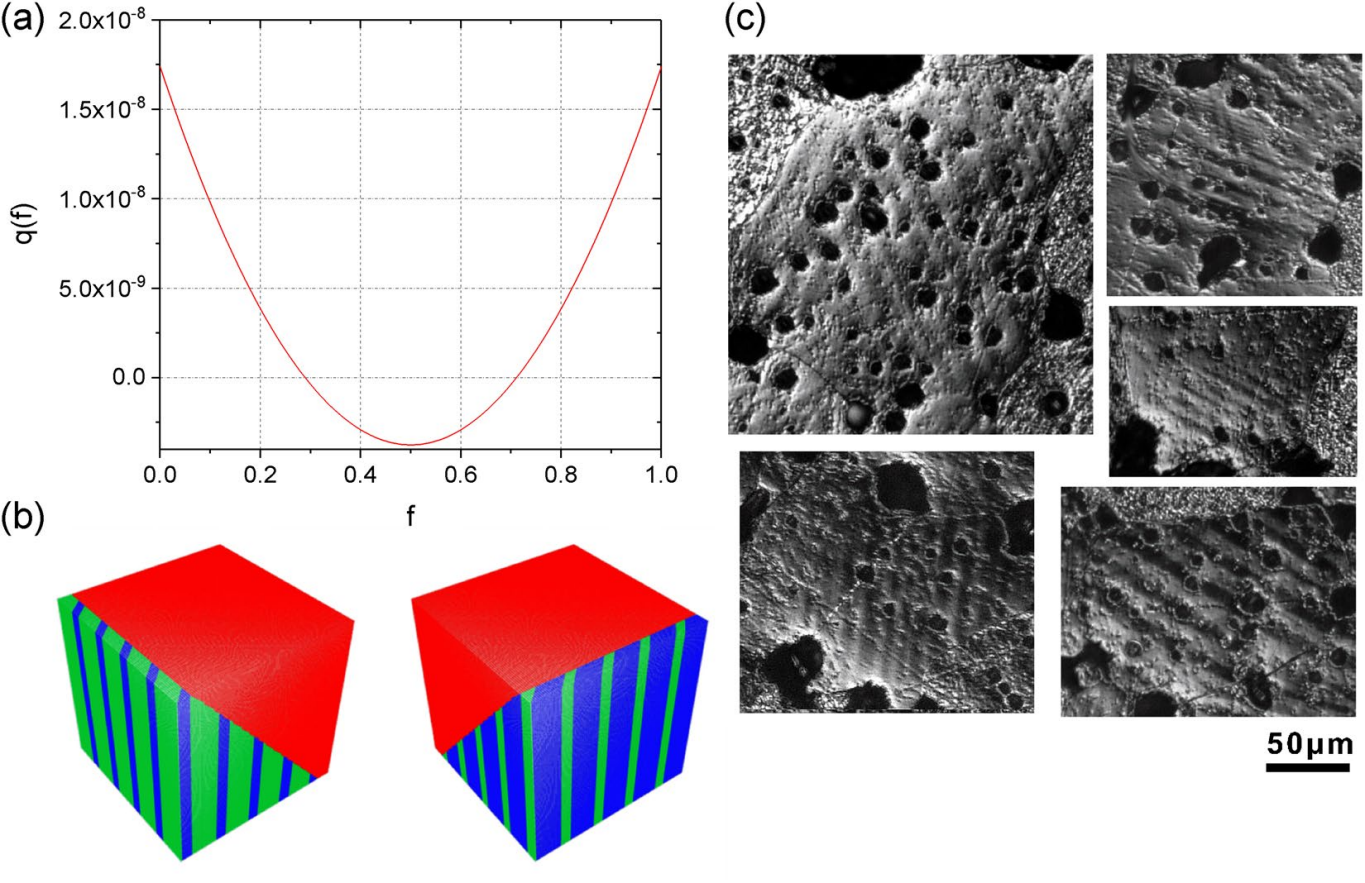
Comparison of theoretically determined structure model of lamellar structure and appearance in the real sample. Graph of (**a**) q(f), (**b**) austenite/martensite interfaces from theory where green and blue areas represent twinned martensitic (i.e., ferroelectric) variants, and red areas represent the austenitic (cubic paraelectric) phase, and (**c**) polarization microscopy images of the investigated twinned domain structure of Ba(Ti_1-x_Zr_x_)O_3_ with x = 0.017 where hysteresis is lowest.

Figure 3a depicts the *q(f)* plot from the measured lattice parameters at the composition x = 0.017 having the lowest hysteresis. This graph stays unaltered no matter which pair of variants is chosen, which is a special situation characteristic of the cubic-to-tetragonal transformation.

There are two observations. First, this is like a classical martensite with two roots. As is known^[10]^ each root gives two distinct solutions of the crystallographic theory. These two solutions of the crystallographic theory are plotted in Fig. 3b. With these small strains the transition layer between the austenitic (paraelectric) phase (see Fig. 3b, red area) and the twinned martensite (ferroelectric) phase (see Fig. 3b, blue and green areas) is present but not visible. Accounting for all possible twin systems in this case there are 24 austenite/martensite interfaces.

The second and more interesting observation is the behaviour of the graph in Fig. 3a. Attention should be drawn to that the scale of the vertical axis, which is extremely small. In fact, by typical measures of satisfaction of the cofactor conditions, this graph is essentially zero. Thus, with extremely small stress this graph could be made identically zero.

We also note that, as *λ*_2_ tends to 1 this graph tends uniformly to 0. However, exactly at *λ*_2_ = 1, the cofactor conditions are not satisfied, because the inequality (CC3) is not satisfied. In summary, at the measured *λ*_2_ for x = 0.017, there are 24 exact and many near austenite/twinned martensite interfaces.

By visualizing the twinned structure by polarization microscopy it was also possible to verify the theoretical value of the volume fraction *f* and *1-f* of the variants. The calculated value for the sample x = 0.017 is *f* = 0.29 (see Fig. 3a), so the volume fraction of the twins should be in the ratio 0.29:0.71. For the bulk sample it was possible to detect the martensitic domain structure with polarized light (see Fig. 3c) and to measure the width of the lamella, which had a mean ratio of 0.64:0.36, which fits nicely to the theoretically expected value.

## Conclusion

The cubic-tetragonal phase transition of the system Ba(Ti_1-x_Zr_x_)O_3_ for x ≤ 0.027 has been characterized regarding change in lattice parameters and the thermal hysteresis. A minimum of the hysteresis *ΔT* and the value of *λ*_2_ closest to one coincide at x = 0.017. The positive influence of the crystallographic compatibility of the two phases on the thermal hysteresis of the phase transition and functional properties like the change of polarization over temperature at the phase transformation which is representative for the electrocaloric effect is noticeable.

## Methods

The Ba(Ti_1-x_Zr_x_)O_3_ ceramics with compositions up to x = 0.027 were prepared by the conventional solid-state reaction technique. The source materials BaCO_3_, ZrO_2_, and TiO_2_ were weighed in a stoichiometric amount and ball milled with addition of hexane with zirconia milling media for 8 h. The powders were calcined two times at 1300 °C for 5 h. After each calcination cycle the samples were again ball milled for 24 h. After drying, the obtained powders were pressed into discs with 20 mm diameter. The addition of polyvinyl alcohol improved the pressing behaviour. The pressed discs were surrounded by BaTiO_3_-granulate during sintering in order to receive a homogeneous temperature distribution. The binder was burnt out at 500 °C for 1 h, afterwards the discs were sintered at 1500 °C for 3 h in air.

The microstructures of the bulk ceramics were characterized by XRD (Rigaku Smart Lab) using a Cu Kα source. For temperature dependent measurement the device was equipped with a heating stage (Anton Paar DHS 1100). The sintered samples were crushed with a mortar for the analysis by XRD in order avoid any stress or texture. The structure analysis was performed by using Rietveld refinement with the software TOPAS-Academic V6 (Coelho Software) with the fundamental parameters approach^29^. Transformation temperatures and latent heats were measured by using a Perkin Elmer Pyris 1 DSC (differential scanning calorimeter) with a heating rate of 10 Kmin^−1^. The weight of the bulk samples was chosen to be in the region of 20 to 25 mg. The transition temperatures were determined with the tangent method. The thermal hysteresis was calculated according to *ΔT *= 1/2(*P*_*s*_ + *P*_*f*_ − *F*_*s*_ − *F*_*f*_).

The composition of the samples was analysed by Laser-Ablation Inductively-coupled plasma mass spectrometry (LA-ICP-MS). The measurements have been performed by measuring against a NIST glass SRM 610 standard. The reference values for this standard were taken from Jochum *et al*.^30^. For electrical characterization the samples were polished and Pt electrodes were applied by sputter deposition. An aixACCT 2000 E TF Analyzer was used to characterize electrical properties. Polarization microscopy images were taken of polished and thermally etched samples in order to visualize domain structures.

## Supplementary information

Supplementary Information.
