# Supplementary material for: Correlation between phase compatibility and efficient energy conversion in Zr-doped Barium Titanate
Source: Sci Rep. 2020 Feb 26;10:3496. doi: 10.1038/s41598-020-60335-5 (PMC7044276; doi:10.1038/s41598-020-60335-5)
Supplement: Supplementary file 1 — Supplementary Information. [file 41598_2020_60335_MOESM1_ESM.pdf]

Correlation between phase compatibility and efficient energy conversion in Zr-doped Barium Titanate

Maike Wegner[1], Hanlin Gu[2], Richard D. James[2], Eckhard Quandt[1,*]

[1] Institute for Materials Science, Kiel University, Kiel, 24143, Germany
[2] Aerospace Engineering and Mechanics, University of Minnesota, Minneapolis, 55455, USA
* eq@tf.uni-kiel.de

Supplementary Information

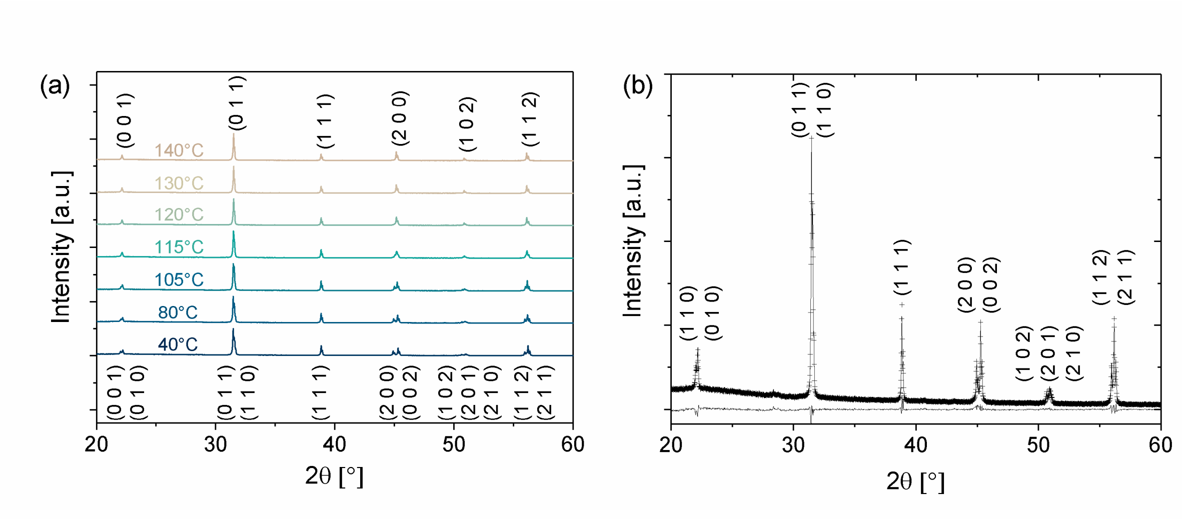

Figure S 1.Temperature dependent XRD measurements and evaluation of data by Rietveld refinement. (a) Example of temperature dependent diffractograms of Ba($Ti_{1-x}Zr_x$)O_3 with x=0.017 at temperatures between 40 °C and 140 °C (b) Structural refinement pattern of Ba($Ti_{1-x}Zr_x$)O_3 with x=0.017 using X-ray powder diffraction data based in the tetragonal phase. Plus (+) marks the observed intensities and the solid line represents the calculated intensities. The difference between observed and calculated pattern is shown beneath.

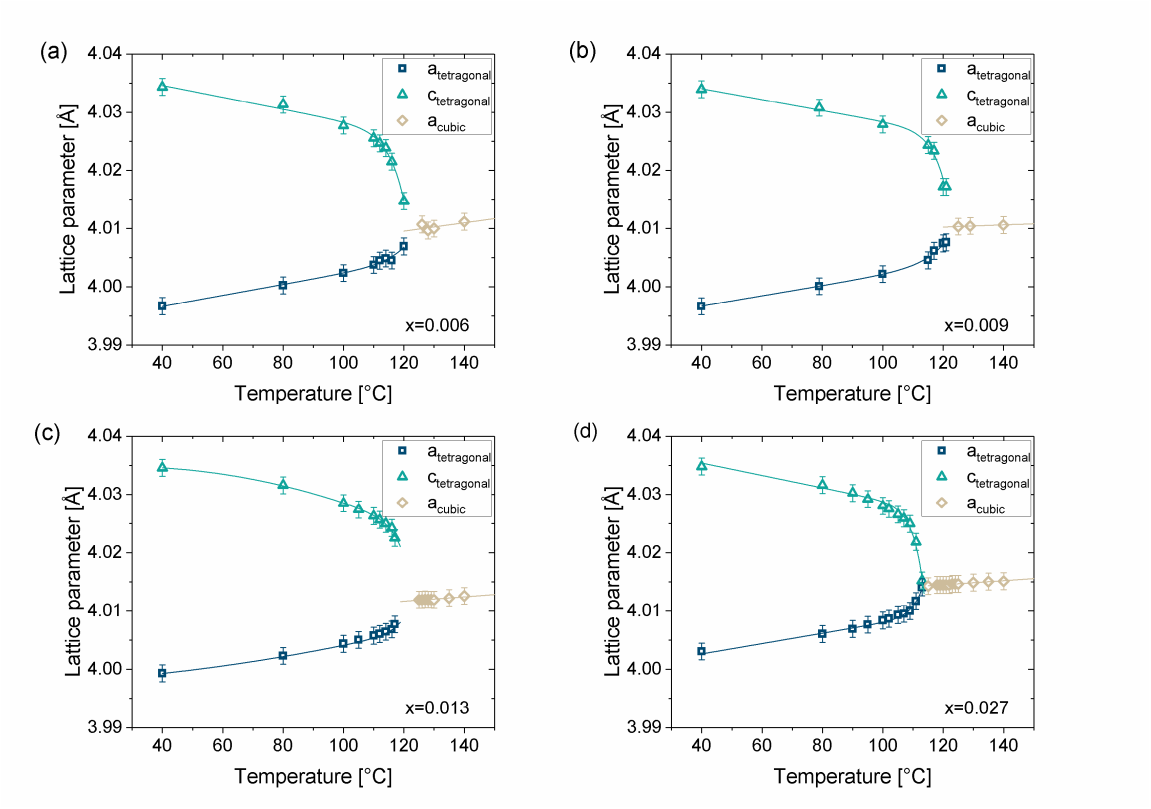

Figure S 2. Temperature dependent lattice parameters of tetragonal and cubic phase for the system $Ba(Ti_{1-x}Zr_x)O_3$ with (a) x=0.006, (b) x=0.009, (c) x=0.013 and (d) x=0.027 with error bars equal to a standard deviation of 0.036 %.

Derivation of the cofactor conditions (CC1), (CC2), (CC3) and the function *q*(*f*)

A brief derivation of the cofactor conditions ((CC1), (CC2) and (CC3) in the text) is given, which explains the occurrence of the function *q*(*f*). Further details and implications of these conditions can be found in the references. [3,22,25]

The cofactor conditions (CC1), (CC2), (CC3) represent a degeneracy of the Crystallographic Theory of Martensite in which there exist infinitely many low energy interfaces between austenite and twinned martensite. The generic case is 4 such interfaces per twin system. (This gives altogether 24 interfaces in the cubic-to-tetragonal case.) Furthermore, when the cofactor conditions are satisfied, many of the new interfaces have zero elastic energy, rather than just low

elastic energy. Supercompatibility refers to the presence of these zero elastic energy interfaces. The usual low energy interfaces have an elastic transition layer whose energy competes with, and balances, the total interfacial energy on the twin boundaries.

The condition (CC1) alone (without (CC2) and (CC3)) is the condition $\lambda_2 = 1$. This is a necessary and sufficient condition that there exists a perfect, unstressed interface between undistorted austenite and a single undistorted variant of martensite. In the cubic-to-tetragonal case this is simply the condition $\lambda_2 = a/a_0 = 1$. This condition arises in the following way. In the cubic-to-tetragonal case we have three variants of martensite described by the three stretch tensors

$$U_1 = \begin{pmatrix} \eta_1 & 0 & 0 \\ 0 & \eta_1 & 0 \\ 0 & 0 & \eta_2 \end{pmatrix}, U_2 = \begin{pmatrix} \eta_1 & 0 & 0 \\ 0 & \eta_2 & 0 \\ 0 & 0 & \eta_1 \end{pmatrix}, U_3 = \begin{pmatrix} \eta_2 & 0 & 0 \\ 0 & \eta_1 & 0 \\ 0 & 0 & \eta_1 \end{pmatrix}, \eta_1 = \frac{a}{a_0}, \eta_1 = \frac{c}{a_0} \quad (1)$$

here written in the orthonormal cubic basis. A perfect interface between the cubic phase and, say, variant 1 of the tetragonal phase is described by the compatibility equation

$$QU_1 - I = a \otimes n \text{ (in components, } Q_{ij}U_{1jk} - \delta_{ik} = a_i n_k) \quad (2)$$

to be solved for the rotation tensor Q and vectors a and n. Here, n is a normal to the interface, a describes the shear of the transformation, and Q describes the rigid rotation of variant 1 needed to secure perfect compatibility with the cubic phase. This equation is solved in reference [24] (Prop. 4). A necessary and sufficient condition that there is a solution Q, a, n is $\lambda_2 = \eta_1 = a/a_0 = 1$; this is the main result used in the text. In the usual case $\eta_2 \neq 1$, there are in fact two solutions.

An equation of the same form as (2) describes the twin boundaries, i.e., the compatible interfaces between variants. For example, variants 1 and 2 have compatible interfaces described by solutions of

$$\bar{R}U_2 - U_1 = c\otimes d \qquad (3)$$

where $\bar{R}$ is a rotation tensor. There are also two solutions $\bar{R}$, c, d of (3) and these exist under the mild condition $\eta_1 \neq \eta_2$. They describe the compound twins of variants 1 and 2. Similar statements can be made about variants 1 and 3 or 2 and 3.

The Crystallographic Theory of Martensite describes low energy interfaces between a twinned laminate (blue and green in Figure 3(b)) and the austenite (red in Figure 3(b)). Supposing that the blue and green are variants 1 and 2, and that equation (3) has been solved so that $\bar{R}$, c, d are known. The deformation of this twinned laminate is described on average by a linear transformation given by

$$H_f = R(f\bar{R}U_2 + (1-f)U_1) \qquad (4)$$

By saying "on average" it is meant that the deformation of the twinned laminate (measured from the cubic phase) is described by the linear transformation H_f except for little zig-zags due to the twins; these zig-zags get smaller and smaller as the twin spacing gets finer and finer, so the deformation of the laminate gets closer and closer to the linear transformation described by (4). In this process of making the twins finer and finer, the volume fraction f of blue vs. green is held constant. With this physical picture in mind it is clear that, if H_f is compatible with I (i.e., the undistorted cubic phase) the elastic energy in the transition layer between austenite and martensite can be made arbitrarily small by making the twins finer and finer. The mathematical condition for this to occur is again a "middle eigenvalue condition", that is, the middle eigenvalue of

$$G_f = H_f^T H_f \qquad (5)$$

is 1. Here ^{T} denotes the transpose. So the problem of the Crystallographic Theory is to find a volume fraction f such that the middle eigenvalue of the positive-definite, symmetric tensor G_f is 1.

To find f such that the middle eigenvalue of G_f is equal to 1, the zeros of $\det(G_f - I)$ have to be found. G_f is clearly quadratic in f and the determinant is a cubic polynomial, so it looks like $\det(G_f - I) = 0$ is a 6th order polynomial equation. But underlying symmetries in the problem[25] imply that it is in fact only quadratic, and, moreover, it is symmetric about $f = 1/2$. The $q(f)$ of the paper is this quadratic function, i.e.,

$$q(f) = \det(G_f - I) \tag{6}$$

The condition (CC2) is the condition that $\frac{dq}{df}(0) = 0$. This, together with (CC1) (which implies $q(0) = 0$) implies that $q(f)$ vanishes identically for $0 \leq f \leq 1$. Wherever $q(f)$ vanishes, *an* eigenvalue of G_f is 1, but it may not be the middle one. (CC3) is necessary and sufficient that, for every zero of $q(f)$, the eigenvalue of G_f that is 1 is in fact the middle eigenvalue. Thus, (CC1), (CC2) and (CC3) are necessary and sufficient that there is a low energy austenite/martensite interface for any volume fraction $0 \leq f \leq 1$.

The scale on the vertical axis of Figure 3(a) shows how near the cofactor conditions are to being satisfied.
